# Phenotypic differentiation of *Streptococcus pyogenes* populations is induced by recombination-driven gene-specific sweeps

**DOI:** 10.1038/srep36644

**Published:** 2016-11-08

**Authors:** Yun-Juan Bao, B. Jesse Shapiro, Shaun W. Lee, Victoria A. Ploplis, Francis J. Castellino

**Affiliations:** 1 W.M. Keck Center for Transgene Research, University of Notre Dame, Notre Dame, IN 46556, USA; 2 Département de Sciences Biologiques, Université de Montréal, Montréal, QC H3C 3J7, Canada; 3 Department of Biological Sciences, University of Notre Dame, Notre Dame, IN 46556, USA; 4 Department of Chemistry and Biochemistry, University of Notre Dame, Notre Dame, IN 46556, USA

## Abstract

Genomic recombination plays an important role in driving adaptive evolution and population differentiation in bacteria. However, controversy exists as to the effects of recombination on population diversity and differentiation, *i.e*., recombination is frequent enough to sweep through the population at selected gene loci (gene-specific sweeps), or the recombination rate is low without interfering genome-wide selective sweeps. Observations supporting either view are sparse. Pathogenic bacteria causing infectious diseases are promising candidates to provide observations of recombination. However, phenotype-associated differentiations are usually vague among them due to diverse disease manifestations. Here we report a population genomic study of the group A *Streptococcus pyogenes* (GAS), a human pathogen with highly recombining genomes. By employing a genome-wide association study on single nucleotide polymorphisms (SNPs), we demonstrate a phenotypic differentiation of GAS, represented by separate clustering of two sublineages associated with niche-specific infections, *i.e*., skin infection and pharyngitis-induced acute rheumatic fever. By quantifying SNPs associated with the differentiation in a statistical and phylogenetic context, we propose that the phenotype-associated differentiation arose through recombination-driven gene-specific sweeps, rather than genome-wide sweeps. Our work provides a novel paradigm of phenotype-associated differentiation induced by gene-specific sweeps in a human pathogen and has implications for understanding of driving forces of bacterial evolution.

A systematic understanding and characterization of the genotype-phenotype correlations in infectious diseases remains a fundamental goal in physiological studies and clinical practices. The correlations are often complicated by multiple host-pathogen interactions, mainly involving versatile adaptive strategies of pathogens through gaining genetic variations to achieve rapid adaptation and prolonged persistence in distinct host environments. The genetic variations include, but are not limited to, point mutations, recombinations, and copy number variations, which in combination result in a spectrum of disease phenotypes for a given pathogen species, leading to clinical complications and difficult diagnostic interpretations.

Over the past two decades, genomic recombination has been found to have a major impact on bacterial evolution, including phenotypic differentiation, ecological cohesion and microorganism speciation^1^,^2^. Multiple models have been proposed to explain the observed patterns of genetic diversity in light of mutation, recombination, and natural selection^3^,^4^,^5^,^6^,^7^,^8^. When recombination events introduce advantageous changes in alleles or genes favored by positive selection, the changes may sweep through the population resulting in reduced sequence diversity within the affected population^5^. The recombination events may also allow neutral or deleterious alleles in proximity to the favorable alleles to “hitchhike” at a high frequency. The sweeps may occur at selected gene loci without affecting the sequence diversity in other regions of the genome, described as gene-specific sweeps^5^,^6^,^9^. When recombination events are frequent across the genome, different loci will support distinct phylogenetic trees, with little support for a single clonal genealogy. Alternatively, in highly clonal bacteria with relatively low rates of recombination, diversity is purged at all loci, described as genome-wide sweeps^10^. Ultimately, the sweeping process at the gene-specific level or the genome-wide scale will imprint specific patterns in the genome, which can be detected by population genomic studies.

The availability of whole-genome information has made it possible to investigate the genetic outcomes of recombinations and the possible evolutionary mechanisms at the level of population genomics based on genome-wide variations. Numerous population genomic studies on genome-wide single nucleotide polymorphisms (SNPs) have demonstrated the profound impact of genomic recombination on driving adaptation and accelerating evolution, such as resistance to antibiotics in *Streptococcus pneumoniae*^11^, association with epidemic outbreaks in *Legionella pneumophila*^12^, and serotype switching in *Chlamydia trachomatis*^13^. Remarkably, genomic recombination has also been shown to induce ecological differentiation *via* gene-specific selective sweeps in aquatic bacteria, such as *Vibrio cyclitrophicus*^14^, *Prochlorococcus*^15^, and *Synechococcus*^16^, and in a soil bacteria population *Mesorhizobium*^17^.

Group A *Streptococcus pyogenes* (GAS) is an obligatory human pathogenic bacterium with complicated colonization environments, ranging from skin surfaces, throat, pharynx, to various deeper internal organs of human body. Clinical manifestations of GAS infection range from benign clinical conditions, *e.g.*, pharyngitis and impetigo, to more lethal outcomes that include necrotizing fasciitis (NF), streptococcal toxic shock syndrome (STSS), and acute rheumatic fever (ARF)^18^. Various clinical strains have been isolated from individual patients or epidemic outbreaks of GAS infection. Many of the causative isolates reemerged with improved fitness or enhanced virulence by acquiring novel genes or large segments of foreign DNA contents *via* genomic recombination^19^,^20^,^21^,^22^,^23^,^24^. GAS has shown remarkable capabilities in rapidly adapting to diverse human body niches and gaining tropism for specific niche environments. The wide range of infection-niche tropism and versatile adaptive capability of GAS have long been recognized to be largely associated with the highly plastic and transformable genome of this organism, as evidenced by frequent genomic recombination events observed in GAS genomes^25^,^26^. However, neither evolutionary mechanisms of the adaptations, nor conclusive associations between the observed infection phenotypes and genotypic properties have been well defined.

Several kinds of genetic markers have been employed to delineate those associations, but have displayed limitations. The *emm* gene locus encoding the surface M protein has been commonly used to serotype GAS strains. Tissue tropism has been based on the peptidoglycan sequences, which define the gene subfamily patterns of the *emm*-like gene locus in the chromosome^27^. However, this method has not been sufficient to account for many of the observed GAS disease phenotypes, including the complex phenotypes of M1 strains^28^, and the multi-tissue tropism exhibited by M3 and M59 strains^24^,^29^. Although the GAS genomes have been shown to be highly recombining^25^, population genomic studies have heretofore not been rigorously applied to GAS in such a manner as to investigate the role of recombination on shaping its mosaic genome architecture and driving its rapid evolution.

In the current study, we conducted a comprehensive population genomic study of natural GAS isolates to elucidate the association of phylogenetic divergence with the infection phenotypes, and to identify possible evolutionary mechanisms underlying the association. We demonstrate a phenotypic differentiation of GAS strains associated with their clinical manifestations, *i.e*., skin infection and acute rheumatic fever (or rheumatogenicity). Using a genome-wide population genomic approach, we further propose that the phenotypic differentiation is induced by gene-specific sweeps driven by genomic recombination. For the first time, we establish an association between genotypes and phenotypes of GAS in causing skin diseases and acute rheumatic fever, and identify the molecular basis of the association in a model of recombination-driven gene-specific sweeps.

## Results

### SNP ascertainment in the core genome of GAS strains

We performed the comparative genomic analysis of the core genomes of 44 distinct GAS strains with known genomic information. As a result, we identified 71,558 SNPs, corresponding to an average of 43 SNPs/kb. This shows a high level of genomic variation compared with the clonal pathogens, such as *Bacillus anthracis*^30^ (0.23 SNPs/kb) and *Mycobacterium tuberculosis*^31^ (6 SNPs/kb). The multi-allelic SNPs were excluded for further study, thus leaving us 68,892 (96.3%) bi-allelic SNPs. Among them, 58,057 (84%) fall into coding regions, and 20,701 (30%) are non-synonymous substitutions causing amino acid changes. The calculation of Watterson’s *θ*^32^ provided a genome-wide average mutation rate of 0.011. This is greater than the average pair-wise nucleotide difference of 0.009, and results in an negative Tajima’s *D* value^33^ of −0.831. This suggests a departure from the neutral evolution expectation of the population of GAS^34^.

### Phylogenetic structure inference reveals population differentiation associated with infection phenotypes of GAS

**Figure 1:**
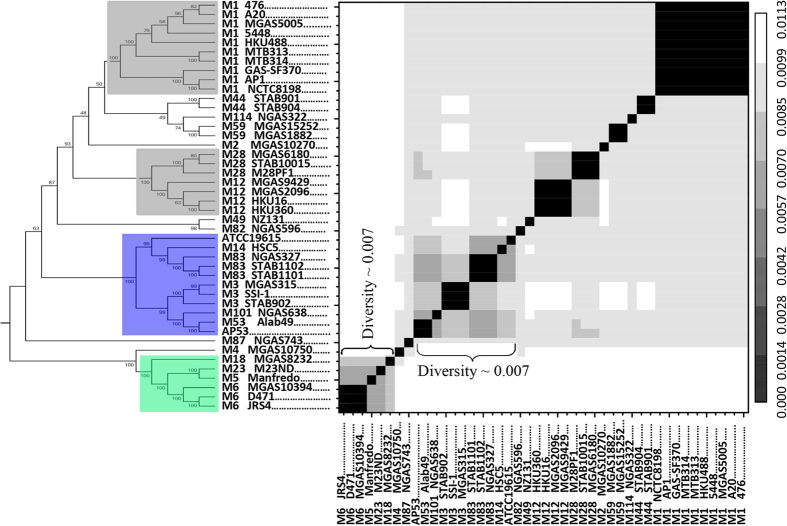
The phylogeny of the core genome of GAS and the heatmap of pair-wise nucleotide differences of the 44 known genomes. The phylogenetic tree was inferred using Maximum Likelihood method with 1000 bootstraps. A grey scale for the nucleotide differences is given on the right side of the heatmap. The population differentiation is clearly observed with high support values, evidenced by the lower nucleotide differences within the clustered strains (~0.007) than the population-wide average (~0.009). The branches of the clustered strains are shaded in color: light blue for strains associated with SI, and light green for those associated with ARF (or rheumatogenicity). The strains from serotype M12 and M28, or M1 are also clustered (in grey shading). However, no further analysis was performed on them due to the limited number of serotypes in those clusters insufficient for statistical tests.

In order to investigate the evolutionary history of GAS genomes, we applied the maximum likelihood method to infer the genome-wide phylogeny based on the detected SNPs. We observed two distinct highly-supported sublineages, with clustered isolates associated with different disease phenotypes depending on the infection niches, *i.e*., skin infection (SI) and acute rheumatic fever (ARF; or rheumatogenicity), respectively (shaded in blue and green in Fig. 1). ARF is a post-infection *sequela* typically developed following throat or pharyngeal infection, mainly affecting connective tissues, such as muscles and joints. The clustering of the strains is further evidenced by the lower within-cluster nucleotide diversity (0.007) than the population-wide average (0.009) (Fig. 1). This demonstrates a phenotype-associated differentiation of GAS revealed by divergent phylogenetic clusters.

GAS has been known to undergo frequent recombination, which interrupts the clonal phylogenetic signals in the genome^25^. Therefore, we examined its haplotype structure by examining the linkage disequilibrium (LD) of the SNP loci using Haploview^35^. Only a limited number of short regions are observed with evidence of strong LD, in comparison with the long-range continuous blocks of LD in the highly clonal species *B. anthracis*^30^ and *M. tuberculosis*^36^ (Supplementary Fig. S1). The highly recombining property of the GAS genomes makes it feasible to perform a genome-wide association study (GWAS) to identify variants associated with niche-specific infections, *i.e*., SI and ARF. In total, we found 895 and 1,638 SNPs associated with SI and ARF, respectively (*p*-value ≤ 10^−3^). Those SNPs cause 221 and 390 amino acid changes in the two sublineages, respectively. The correction for population stratification was performed by excluding the strains with redundant M serotypes from the test due to the close phylogenetic relationship between them. The Q-Q plots show relatively weak inflation in the association test (the inflation factor λ = 1.07~1.34) (Supplementary Fig. S2). Further analysis with the Cochran-Mantel-Haenszel (CMH) test does not improve the inflation factor, implying that the influence of population stratification on the association test has been well controlled by excluding redundant M serotype strains from the test. However, we note that the permutation testing for multiple corrections only leaves no more than 50 candidates with a corrected *p*-value ≤ 0.05, probably due to the small sample size and unequal proportion of associated and non-associated samples. Therefore, for further study, we chose SNPs with significant non-corrected *p*-values ≤ 10^−3^ and high odds ratios ≥ 28 by fitting the overall distribution of the *p*-value and odds ratio (Supplementary Fig. S3). Due to the genomic inflation, some of these SNPs are likely false positives. Therefore, we did not focus on any particular SNPs in detail, but rather focus on the whole set of associated SNPs that are also enriched in true positive associations.

### Phenotype associated SNPs predominantly arise from gene-specific sweeps

**Figure 2:**
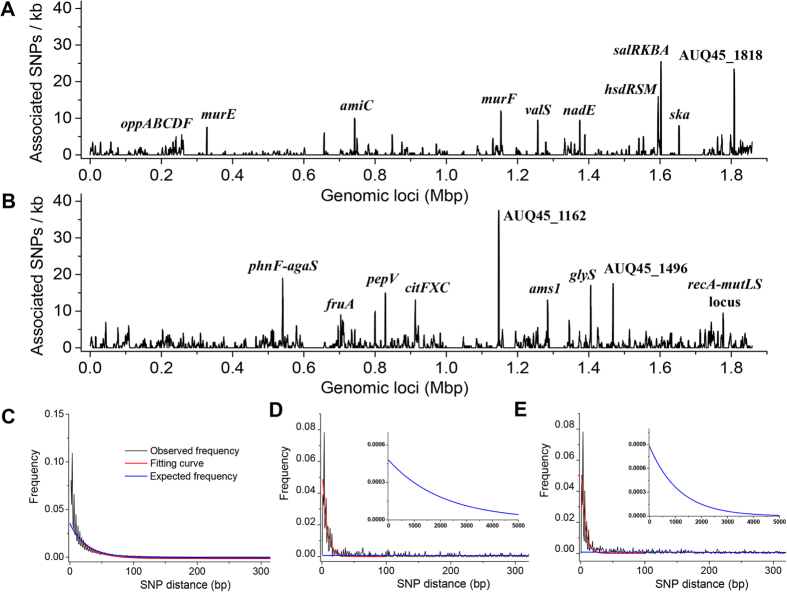
Highly concentrated distributions of the SNPs associated with niche-specific infection. (**A**,**B**) Spatial density of SNPs associated with SI and ARF, respectively. The gene loci harboring the most significant clustering of SNPs are indicated and represented as high peaks in the density. (**C**–**E**) Distribution of inter-SNP distances for genome-wide SNPs, SNPs associated with SI, and those associated with ARF, respectively. The observed distribution was fitted and compared with the expected exponential distribution, assuming a random independent occurrence of the SNPs. The fitting curve for the genome-wide SNPs is consistent with the expected distribution. However, the fitting distribution for SNPs associated with SI or ARF deviates from the expected distribution, revealing excess of closely located SNPs. The inset in (**D**,**E**) shows the zoomed curve of the expected distribution.

We propose that the phenotype-associated differentiation of the GAS population is induced by gene-specific sweeps, based on four lines of evidence. First, the non-random spatial distribution of the associated SNPs forms concentrated clusters at several genes, gene operons, or neighboring genes, based on a sliding window analysis (Fig. 2A,B). The clustering of the SNPs indicates the dependence among neighboring SNPs, which could possibly be brought about by recombination. To test the dependence between SNPs, we compared the distribution of the observed inter-SNP distances against those that are expected assuming a random independent occurrence of SNPs across the genome. The fitting curve for all genome-wide SNPs is highly consistent with the expected exponential distribution (Fig. 2C). In contrast, the fitted distributions for SNPs associated with SI or ARF deviate from those expected, manifested by an excess of closely located phenotype-associated SNPs (Fig. 2D,E). This reveals a pattern predicted by gene-specific sweeps, with frequent recombination and positive selection on the phenotype-associated SNPs.

**Figure 3:**
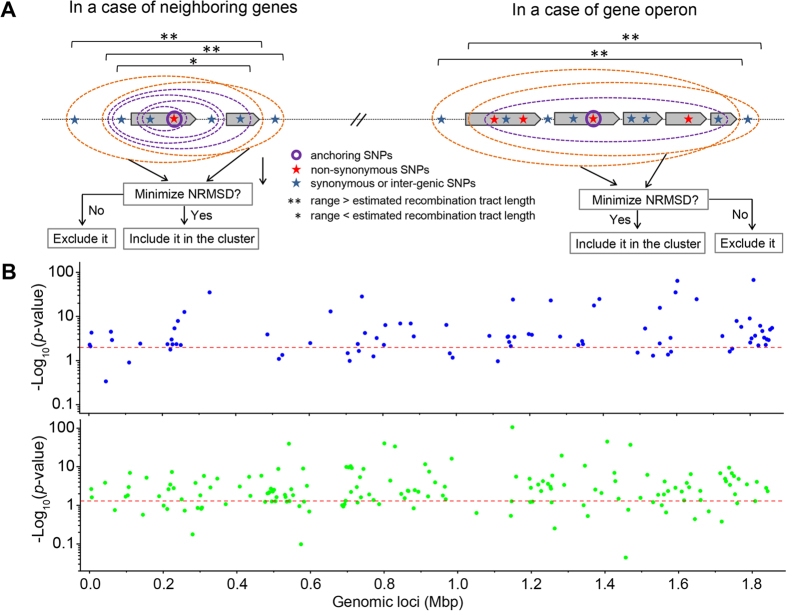
Assessment of clustering of SNPs associated with niche-specific infection. (**A**) Illustration of the anchor-extension strategy for categorizing the associated SNPs in clusters based on a model of gene-specific sweeps. A non-synonymous SNP is first anchored as an initial cluster and the initial cluster is extended by including the neighboring SNPs within a range of estimated recombination tract length L. If the SNPs fall into a single gene or gene operon of length longer than L, all the associated SNPs in that gene or gene operon were included in the target cluster. The extension of the target cluster is indicated by color coded ovals, with purple for a cluster spanning a distance shorter than L or within a gene operon, and orange for a cluster spanning a distance longer than L or beyond a gene operon. The inclusion of ambiguous SNPs on the boundaries was determined by minimizing the normalized root-mean-square of inter-SNP distances NRMSD. (**B**) Manhattan plots of the significance of SNP clusters. The significance is evaluated against a model of independent and random distribution of SNPs across the genome (top panel for SI and bottom panel for ARF). The red dotted lines show the threshold for statistical significance (*p*-value ≤ 0.01 for SI; and *p*-value ≤ 0.05 for ARF).

The second line of evidence is based on the significance of the clustering and the composition of the clusters. Under a model of gene-specific sweeps, non-synonymous SNPs under selection would be surrounded by clusters of non-selected SNPs (*i.e*., synonymous SNPs and inter-genic SNPs), which may hitchhike in the same recombined tract of DNA. In order to assess the significance of the SNP clustering, we first quantified the associated SNPs into spatially discrete clusters (≥2 SNPs per cluster) by simulating the process of gene-specific sweeps. To accomplish this goal, we employed an anchor-extension strategy based on a non-synonymous SNP-centric concept (Fig. 3A): anchoring an initial cluster at non-synonymous SNPs (assuming that the non-synonymous SNPs are more likely to be positively selected) and extending the initial cluster on both sides within a range of the estimated recombination tract length, L. The significance of the clustering was subsequently assessed by calculating the probability of the SNPs in each cluster located within the L range under a model of independent and random distribution of SNPs across the genome. Ultimately, the majority of the non-synonymous SNPs (>89%) fall into 86 (SI) and 159 (ARF) well-defined clusters characteristic of gene-specific sweeps. Among the defined clusters, 68 (79%; SI; *p*-value ≤ 0.01) and 114 (72%; ARF; *p*-value ≤ 0.05) are significant (Fig. 3B and Supplementary Dataset 1). These clusters are composed of non-synonymous SNPs and predominantly synonymous SNPs in the same genes/gene operons (or neighboring genes), but are also partly inter-genic SNPs of nearby regions. This suggests hitchhiking of synonymous or inter-genic mutations with positively selected non-synonymous mutations. Alternatively, each cluster of synonymous, non-synonymous, and inter-genic mutations could have been introduced at the same time by a single recombination event from a distant relative, which subsequently swept through the sublineages.

**Figure 4:**
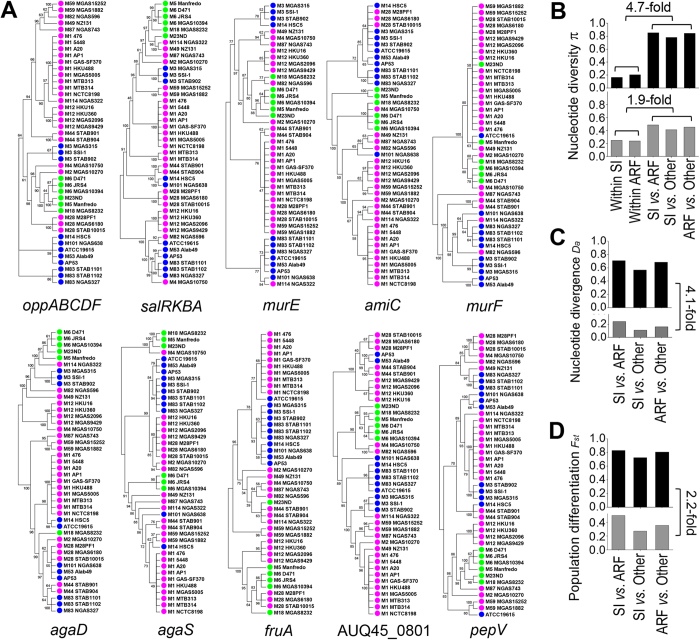
Phylogenetic incongruence of phenotype-associated gene loci and alterations of nucleotide diversity of those gene loci. (**A**) Phylogenetic trees reconstructed for SNP loci flanking the associated SNPs at different gene loci. Nineteen of the gene loci with the most significant SNP clustering were selected and the trees for ten of them are shown (see Supplementary Fig. S4 for the full set of trees). All trees exhibit topological deviation from the core genome phylogeny and each tree is topologically distinct. The nodes in the trees are indicated in color: blue for the sublineage associated with SI, green associated with ARF, and magenta for the remaining strains in the population. The support values of the trees were derived by bootstrapping 1000 replicates. Only the topology of the trees is shown by ignoring the branch lengths. (**B**–**D**) Alterations of genetic diversity of the associated SNPs (top panel in black bars) in comparison with those flanking the associated SNPs (bottom panel in grey bars). Three quantities are shown: nucleotide diversity π between sublineages and within sublineages (**B**), nucleotide divergence *D*_*a*_ between sublineages (**C**), and population differentiation *F*_*st*_ between sublineages (**D**). The ratio of inter-sublineage *versus* within-sublineage nucleotide diversity shows substantial decrease for the flanking non-associated SNPs compared with the associated SNPs. Similar decrease is also observed for nucleotide divergence and population differentiation.

Our third line of evidence for gene sweep-induced differentiation is suggested by the phylogenetic incongruence of different genes across the genome. By definition, the phenotype-associated SNPs result in a gene tree supporting the separate grouping of the strains associated with SI and ARF. Under a genome-wide sweep model, the rest of the genome, including genes flanking the associated SNPs, would follow the same clonal genealogy. Under the gene-specific sweep model, different flanking regions would support distinct phylogenies, possibly with SI and ARF grouped separately. To distinguish between the two models, we selected 9 and 10 of the most significant SNP clusters associated with SI and ARF, respectively. We next extracted the neighboring SNP loci and constructed the phylogenetic trees from these loci. All of the reconstructed trees show topological deviation from the core genome phylogeny. This supports monophyletic clustering of the strains associated with SI and ARF (Fig. 4A and Supplementary Fig. S4). Specifically, only three out of the 19 trees support the clustering of strains associated with SI and six of the trees support the clustering of strains associated with ARF, and, remarkably, none of the trees supports the separate clustering of the two groups.

Furthermore, we also observed tight linkage of the phenotype-associated SNPs in clusters, as represented by the localized continuous LD blocks, indicating the simultaneous introduction of the SNPs within clusters *via* recombination or hitchhiking with gene sweeps (Supplementary Fig. S5). We observe that the tight linkage is interrupted by the nearby SNPs surrounding the clusters, which is manifested by the shorter and fragmented LD blocks (Supplementary Fig. S5). This is again consistent with phenotype-associated SNPs spreading through the populations *via* gene-specific sweeps, resulting in linkages within the associated SNP clusters but little linkage of these clusters and the rest of the genome.

Under the gene-specific sweep model, the phenotype-associated genes should be highly differentiated between sublineages with different phenotypes, and should contain relatively low diversity within sublineages. Consistent with the gene-specific model, we observed that the associated SNP loci show high differentiation between the sublineages and low diversity within each sublineage (Fig. 4B–D). Specifically, the inter-sublineage nucleotide diversity is on average 4.7-fold higher than the within-sublineage when considering the associated SNP clusters. For those nearby SNPs surrounding the clusters, the difference is decreased to 1.9-fold (Fig. 4B). The inter-sublineage divergence (*D*_*a*_) and differentiation (*F*_*st*_) also show substantial decreases when considering the nearby SNPs surrounding the clusters by an average of 4.1-fold and 2.2-fold, respectively (Fig. 4C,D). These patterns are consistent with the gene-specific sweeps, which purge the genetic variations at specific gene loci under positive selection, while maintaining the diversity in other regions of the genome.

### Recent recombination events in GAS genomes are more prominent within sublineages than between sublineages

In order to quantify the impact of recombination events, we first assessed the intragenic conversions of DNA fragments within each coding gene between pair-wise strains using geneconv^37^ and the Phi test^38^. A total of 106 out of the 767 genes included in the analysis (containing at least 10 SNPs per gene) carry significant signals of recombination between at least one pair of strains (Table 1). Two features are observed for the recombination signals among the 106 genes: (i) A shorter mean fragment length is observed for intragenic recombination between sublineage SI and ARF (389 bp) than within each of the sublineages (554 bp and 525 bp, respectively) (Table 1); (ii) Significantly more recent recombination events were detected within the sublineage SI than between the sublineages SI and ARF (*p*-value = 0.0007; Fisher’s exact test) by comparing the number of recombination fragments within sublineages and between sublineages (Table 1 and Supplementary Fig. S6). The significance test suggests that the recent intragenic recombination is more prominent within sublineages than between sublineages, probably due to the higher sequence divergence between sublineages^6^,^14^. The recombination between sublineages probably represents more ancient events that occurred before the separation of the sublineages, and has eroded gradually, resulting in shorter and fewer recombination fragments to be detected. Figure S7 shows the recombination events in two representative genes *valS* (valyl-tRNA synthetase) and *lacE* (lactose-specific PTS system IIB/IIC component) with more recombinations within the sublineage SI than between the sublineages SI and ARF. We generally observed that the occurrence of recombination is negatively correlated with sequence divergence between the sublineages.

### Relative contributions of recombination and mutation in shaping the population structure

Our findings thus far strongly suggest that phenotypic differentiation of the GAS genomes occurred through acquisition of infection niche-specific SNPs predominantly *via* genetic recombination. Next, we sought to quantify the relative contributions of recombination and point mutation (r/m) to differentiation in the GAS genomes. We estimated the ratio r/m by comparing the number of non-synonymous associated SNPs acquired through recombination against point mutations. We only used the non-synonymous SNPs for the estimation of r/m considering that the synonymous SNPs or non-coding SNPs are probably more likely to be hitchhiked and less likely to be selected, and therefore may bias the estimation, although we also note that recent studies showed that the sequence variations in non-coding regulatory regions may also result in fitness effects^39^,^40^. The non-synonymous point mutation candidates were identified as singleton associated SNPs, which cannot be categorized to any SNP cluster and are unlinked to the neighboring SNP clusters. We obtained 10 and 12 such point mutations associated with SI and ARF, respectively (Supplementary Table S1), corresponding to the ratio r/m of 22~34. This approach represents a conservative estimate, considering that the SNPs outside the clusters may also arise from recombination events, which are nevertheless difficult to unambiguously identify. This empirically derived ratio is comparable to that estimated by ClonalFrame^41^, which gave a median r/m of 34, from averaging over 15 randomly selected genomic regions (Supplementary Fig. S8 and Table S2). This suggests that more than 90% of the polymorphisms in GAS genomes may have been introduced by recombination and clearly demonstrates the remarkable impact of genetic recombination on driving adaptation and diversification of the genetic repertoire.

### Functional implications of the phenotype associated SNPs

We found high representation of the associated SNPs in genes involving functions of basic molecular metabolism and membrane transport (Table 2). These functions reflect the deterministic factors in adaptation for GAS in human niches, *i.e*., nutrient utilization and molecular metabolism, for optimizing intracellular growth and survival. A similar result was also reported in the analysis of the population variations in a human cavity-causing bacterium, *S. mutans*, where the genes associated with adaptation to, or benefits from, a human oral niche are mainly involved in carbohydrate metabolism, metal and peptide translocation, oxidative stress response, and others^42^. Interestingly, we observed that the functional category, resistance to antibiotics and toxic compounds, is more represented in strains associated with ARF (*p*-value ≤ 0.05; Fisher’s exact test). Such allele changes may reflect the molecular response of the bacteria in combating the specific challenges they encounter in causing ARF. The SNPs associated with SI and ARF are both enriched in the gene locus comprising the *recA* and *mutL*-*mutS* system, which are responsible for genetic recombination and DNA mismatch repairs. Multiple non-synonymous mutations were found in the gene *cinA* within the locus, which is proposed to be specifically required at some stage of genetic transformation. This further supports the importance of the high rate of genetic recombination in GAS in driving phenotypic differentiation and evolution.

### Phylogenetic structure of the accessory genome differs from the core genome phylogeny

**Figure 5:**
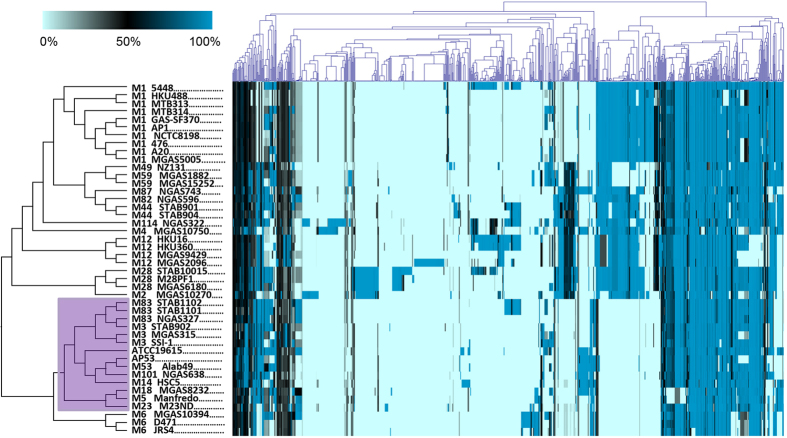
The phylogenetic tree of the accessory genome of GAS and the clustering of accessory genes. The tree was derived based on a hierarchical clustering analysis of the pair-wise homology of accessory genes between the GAS genomes. The tree for the GAS strains is shown on the left and the clustering for the accessory genes on the top. The heatmap indicates the homology of pair-wise flexible genes between GAS genomes. A color key is shown on the top left. The accessory gene tree is topologically different from the core genome phylogeny. Three of the strains associated ARF or rheumatogenicity (M18 MGAS8232, M5 Manfredo, and M23ND) cluster together with the strains associated with SI (shaded in purple).

We constructed the phylogeny tree for the accessory GAS genomes by identifying the dispensable genes, which are partially encoded by GAS genomes (Fig. 5). Neither the overall topology of the tree, nor the ecological differentiation associated with niche-specific infection, is maintained as strongly as observed for the core genome (Fig. 1). The alteration of the phylogeny indicates the distinct evolutionary history of the accessory genome from the core, probably shaped by recombination *via* horizontal gene transfer. It is further evidenced by the substantial sequence mosaicism and divergence in the accessory genes. As much as 89% of the accessory genes are completely absent in at least one of the genomes, and 15% of them are specific for individual strains. Specifically, the strains from the sublineage SI and ARF cluster together, forming a monophyletic branch. Furthermore, three of the strains associated with ARF (or rheumatogenicity), *i.e*., M18 MGAS8232, M5 Manfredo, and M23ND, are more closely related with the sublineage SI (shaded in purple in Fig. 5). These findings indicate that gene content exchanges in the accessory genome between the two sublineages occurred. Therefore, we performed gapped multiple sequence alignment of the accessory genome and calculated pair-wise nucleotide diversity between the GAS genomes. The calculation shows that there is no significant difference in the sequence diversity within and between the sublineages SI and ARF (*p*-value > 0.07, *t*-test), indicative of the absence of complete genetic barriers to horizontal gene transfer between the two sublineages. A prominent example for horizontal gene transfer between the sublineages SI and ARF is the FCT locus (encoding Fibronectin-binding proteins, Collagen-binding proteins and T-antigens), where several strains associated with SI and ARF share the same pattern of gene organization^43^. The surface proteins encoded by the locus facilitate the adherence of bacteria to host cells, and the host cell surfaces in turn may provide venues for the bacteria to disseminate gene contents between the strains.

## Discussion

With the widespread availability of genomic information, one of the fundamental goals has been to delineate the associations between genetic characteristics and clinical phenotypes. The associations are complex and in many cases are vague, especially in infectious diseases caused by bacterial pathogens, where phenotypic and genotypic heterogeneity make such systematic inferences difficult. In the versatile human pathogen, Group A *Streptococcus pyogenes* (or GAS), the heterogeneity is largely facilitated by the highly recombining genomes of the organism, where stretches of DNA fragments are exchanged or transferred within the bacterial community or across distant species. A population genomic study of GAS genomes based on genome-wide SNPs demonstrated an explicit differentiation in the phylogenetic structure associated with niche-specific infections, *i.e*., skin diseases and pharyngeal-induced acute rheumatic fever (or rheumatogenicity). Although ecologically-associated population differentiation has been observed in aquatic and soil bacterial organisms^14^,^15^,^16^,^17^, ours is the first such report demonstrating gene-specific sweeps driving infection-niche tropism in a human pathogen such as GAS, a species with multiple clinical outcomes and diagnostic complications. We observed that the phylogenetic clustering from SNPs in our study is different from that based on the commonly used genetic markers, such as the *emm* pattern. For example, serotype M3 and M14 strains have long been classified as *emm* pattern A-C, which is proposed to be more closely related with pharyngeal infection. In contrast, these strains are clustered together with the skin-tropic strains with emm pattern D in the current study. This may explain why the isolates from M3 and M14 also caused skin-associated diseases, such as necrotizing fasciitis or streptococcal toxic shock syndrome. It is possible that the two types of strains have changed their serotypes by undergoing distinct recombination in the *emm* gene locus, or they have become rapidly adapted to skin niches by acquiring allele changes at specific gene loci.

Further statistical tests of the SNPs associated with the infection phenotypes reveal that those associated variations predominantly arise from gene-specific selective sweeps *via* recombination. This is supported by: (i) the associated SNPs are not randomly distributed, but clustered at several gene loci, with the fitted distribution significantly deviating from the expected distribution in a model of random independent occurrence of the SNPs. (ii) Most of the associated SNPs fall into statistically significant clusters, comprising of non-synonymous SNPs, and predominantly synonymous SNPs in the same genes/gene operons (or neighboring genes), but also partly inter-genic SNPs of nearby regions, characteristic of hitchhiking of synonymous or inter-genic SNPs with positively selected non-synonymous SNPs. (iii) Incongruence of the phylogenetic trees is observed for the nearby SNPs not associated with infection phenotypes compared with the associated SNPs, suggesting the distinct evolutionary processes in the swept regions and the rest of the genome. (iv) There is substantial difference in the population diversity parameters for the associated SNPs as compared with the non-associated nearby SNPs, *i.e*., higher ratio of inter-sublineage diversity to within-sublineage diversity (from 4.7 fold to 1.9 fold), signifying the effects of gene-specific sweeps on purging the variability out of the subpopulation at specific gene loci while maintaining the diversity in other regions of the genome. Overall, the genetic characteristics strongly suggest the phenotype-associated differentiation is predominantly induced by recombination-driven gene-specific sweeps. Compared with the low rate of point mutations, the rate of recombination is sufficiently high (22–34:1) such that the gene sweeps occur rapidly for the bacteria to become adapted to diverse host environments. The selected gene loci were homogenized by the sweeps within the associated sublineages, resulting in a cohesively adaptive population while maintaining the population-wide divergence^3^,^4^,^5^. The homogenization of gene sweeps within sublineages has two effects in the GAS genomes: (i) The recent recombination events occur more prominently within sublineages than between sublineages (*p*-value = 0.0007), due to reduced sequence divergence within sublineages; (ii) There is a shorter recombination length between sublineages than within sublineages. This implies that the recombination events occurring between sublineages are more ancient than those within sublineages and has eroded during the course of its evolution. The recombination events that occurred within sublineages, which have largely shaped the current population structure, may have just recently emerged. It is consistent with the concept that GAS is an obligatory human pathogen, and its adaptation to the human host is relatively recent on the scale of its evolutionary development.

Even with the overwhelming contribution of recombinations *versus* point mutations to genomic variation (>90%), the true level of recombination in GAS has been very likely underestimated due to the limited sample size, or the undetectable events, involving the sequences from distant taxa outside of the population. Moreover, the use of SNP data for the study of recombination prevents us from detecting gene gain and gene loss events, such as phage acquisition, which has been extensively studied in GAS^21^,^44^,^45^,^46^,^47^,^48^. On the other hand, the inherent nature of hitchhiking in the gene sweeps implies that not all of the allelic changes introduced from gene sweeps will carry benefits for the adaption and survival of specific lineages. Therefore, the actual number of selective variations relevant to niche-specific infection could be different from that identified in the recombined regions mediated by gene-specific sweeps.

The associated SNPs are highly represented in genes involving functions of basic molecular metabolism and membrane transport for both sublineages associated with SI and ARF. This may reflect the common functional aspects in the micro-adaptation process for the bacteria to circumvent diverse challenges in human environments. With regard to the differential representation of the functions between the two sublineages, we observed that the SNPs involved in resistance to antibiotics and toxic compounds are significantly more represented in ARF (*p*-value ≤ 0.05). Interestingly, we note that the isolates causing ARF emerged shortly after the use of antibiotics for treatment of ARF^49^ or in a recurrent epidemics of ARF^50^. Recently, reports of ARF development following macrolide treatment failure in GAS pharyngitis due to macrolide resistance have been recorded, suggesting a possible link between our observation and clinical diagnoses, though further studies are needed in this regard^51^.

Our study provides the first paradigm of disease phenotype-associated differentiation in a human pathogen causing clinical complications, which to date has lacked robust identification of genotype-phenotype associations. Using a population genomic approach, we reveal a phenotypic differentiation of GAS genomes associated with two niche-specific infections, *i.e*., skin diseases and acute rheumatic fever (or rheumatogenicity). Based on statistical tests, we propose the molecular basis of the differentiation attributed to recombination-driven gene-specific sweeps. However, it should be noted that our study is based on a limited sample size, and may have lacked power to identify the genotype-phenotype associations in a comprehensive manner. Additional sampling of strains with contrasting phenotypes from multiple geographical regions and time points would likely increase the power to detect the associations.

We observed that the mechanism of the phenotypic differentiation of GAS in the current study parallels that of the ecological differentiation of a marine bacterium *Vibrio cyclitrophicus*^14^ and soil bacteria *Mesorhizobium*^17^ with respect to the gene-specific sweeps. It is intriguing that the genetic mechanism for ecological differentiation is shared between environmental bacteria and human adapted pathogens, suggesting that the gene-specific sweep may represent a common mechanism of niche adaptation for bacterial organisms.

## Methods

### SNP detection and estimation of genetic parameters

The genomic sequences for the GAS strains were obtained from the NCBI Genbank database. The core genome was created by aligning the fragmented genomes against one of the complete genomes AP53, which was sequenced and investigated by our group previously. The mosaic regions with ambiguity in determining SNPs were excluded, including the repetitive regions, phage elements, and transposons. The polymorphisms were detected using VAAL^52^. The Watterson’s *θ* was calculated based on the segregating site estimator as defined by Watterson^32^. The average pair-wise nucleotide difference was calculated by counting the number of nucleotide differences for each pair of genomes normalized to the core genome length. Tajima’s *D* test of neutrality was calculated as defined by Tajima^33^. The calculation of Watterson’s *θ* and Tajima’s *D* was performed on 21 strains containing non-redundant M serotypes to avoid biased estimation due to the low sequence diversity between strains with the same serotypes.

### Genome-wide association analysis of SNPs associated with phenotypic differentiation

The genome-wide association test was performed using the Chi-squared test. In order to reduce the influence of the population structure on the association test, we excluded the strains with redundant M serotypes from the analysis due to the close relationships between them. The inflation factor, λ, of the association test was calculated as the ratio of the observed median Chi-square and the expected median Chi-square under the hypothesis of no association. Correction for population stratification was performed using the Cochran-Mantel-Haenszel (CMH) test with no improvement on the inflation factor, implying that the influence of population stratification has been well controlled by excluding strains with redundant M serotypes from the association test. The multiple testing corrections using permutation testing only leaves no more than 50 candidates with a corrected *p*-value ≤ 0.05, and therefore we chose the SNPs based on the overall distribution of non-corrected *p*-value and odds ratio (Supplementary Fig. S3). The final set of significant SNPs has *p*-values ≤ 10^−3^ and odds ratios ≥ 28.

### Sliding window analysis of the spatial SNP density

The SNP density was calculated by counting the number of SNPs in a sliding window of 2000 bp. The density was normalized to every 1000 bp. Several regions show significantly higher SNP density than the average.

### Fitting the distributions of inter-SNP distances

Assuming that the position of mutational events is a random variable, the distribution of SNPs can be described using a Poisson process model and the distance between SNPs should follow the exponential distribution^53^:


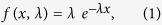


where λ is the mutation rate. When the recombination rate is non-zero, the distribution of SNP distances can be generalized to the gamma distribution^54^:


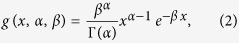


where α determines the shape of the gamma distribution and β is the mutation rate. The observed distribution of distances for the genome-wide SNPs was well fitted to a gamma distribution with α = 1, β = 0.037, which is coincident with the exponential distribution with a mutation rate of λ = 0.037. However, the distance distribution for SNPs associated with SI and ARF are fitted to a variable of the exponential distribution:


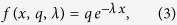


where 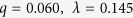 for SI-associated SNPs, and 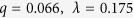 for ARF-associated SNPs, deviating from the expected exponential distribution with 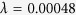, and 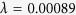, for SI and ARF respectively. The parameters of the distribution were determined based on Chi-squared minimization.

### Clustering of SNPs associated with phenotypic differentiation and the significance test

The SNPs identified to be associated with ecological differentiation were grouped into spatially discrete clusters in order to examine the presence of inter-dependence of SNPs characteristic of gene-specific sweeps. The grouping was performed with an anchor-extension strategy based on a non-synonymous SNP-centric strategy: a non-synonymous SNP with association was first anchored as an initial cluster and then the initial cluster was extended by scanning the neighboring associated SNPs within a range of 1,781 bp, which is the estimated recombination tract length L. If the SNPs fall into a single gene or gene operon of length longer than L, the scanning of the neighboring SNPs was performed in the whole range of the gene or gene operon. The inclusion of ambiguous SNPs on the boundaries was determined by minimizing the normalized root-mean-square of the inter-SNP distances in the target cluster and manually inspected:


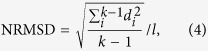


where *d*_*i*_ is the i^th^ inter-SNP distance, *k* is the total number of SNPs in the target cluster, and *l* is the spanning length of the SNPs in the cluster. Each cluster contains at least 2 SNPs. The significance of the SNP clustering was evaluated conditioning on a null hypothesis that the mutations are independently and randomly distributed across the chromosome. Under the null hypothesis, the probability that 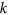 distinct SNPs clustered within a distance of *l* can be estimated based on a gamma distribution given an average mutation rate of μ^55^:


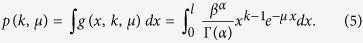


### Calculation of genetic diversity and population differentiation

The calculation of nucleotide diversity π (the average number of nucleotide differences per site between two individuals) and nucleotide divergence *D*_*a*_ (the average number of net nucleotide differences per site between two sub-populations) was determined according to the definition by Nei and Li^56^. The analysis of population differentiation *F*_*st*_ was based on an estimator by Hudson *et al*.^57^. In order to remove the bias induced from the closely related strains with the same M serotypes, the strains with redundant M serotypes were excluded from the calculation.

### Reconstruction of the phylogeny structure

The core genome phylogenetic structure was inferred using Maximum Likelihood method implemented in MEGA^58^ with 1000 bootstraps based on the concatenated alleles at all SNP loci and coding SNP loci, respectively. The trees from both sets of SNPs show no differences. The accessory genome tree was constructed using hierarchical clustering analysis of pair-wise homology of accessory genes between GAS strains. The accessory genes were identified based on reciprocal BLAST comparison with homology lower than 50% for any compared orthologs. The sequence diversity of the accessory genome was estimated as the number of nucleotides absent/present between each pair of genomes based on multiple gapped alignment of the GAS genomes with the core genome removed using Mauve^59^. In order to avoid the overwhelming influence of phage elements and transposons on the accessory genome quantification, the phage sequences and transposons were also removed in the construction of phylogenetic trees and sequence diversity calculation. For constructing trees for protein-coding genes or SNP loci flanking the SNP clusters, the non-informative SNPs (those SNPs that only occur in one of the strains) were excluded. The topological conflicting between trees was measured using the path difference metric, which calculates the differences in the lengths of the paths for each pair of tree leaves. The calculation was implemented in the R environment.

### Detection of intragenic recombination and identification of recent recombination events

The intragenic recombination in protein-coding genes was detected with geneconv^37^ and PhiPack^38^ based on a gene conversion model of recombination. Only the genes containing at least 10 SNP loci were considered, leaving us a total of 767 qualified genes. The multiple sequence alignments for each of the 767 genes among the GAS genomes were constructed and subsequently subject to recombination detection. The recombination fragments were considered to be significant if the Bonferroni-corrected KA *p*-value ≤ 0.05 in geneconv and the *p*-value ≤ 0.05 for at least two of the three methods (Phi permutation, Neighbor similarity score and Max χ^2^) implemented in PhiPack. The potential recent recombination events are defined as those that involve gene fragments that have a tree distance shorter than a specified threshold compared to the core genome phylogeny. The threshold was determined based on the overall distribution of the tree distance assuming that recent recombination should not severely affect the phylogeny structure shaped by selective sweeps. The significance test of the occurrence of recent recombination events was performed within the sublineage SI, but not within ARF due to the smaller number of samples in this sublineage.

### Estimation of the relative contribution of recombination to mutation

The empirical estimate of the recombination to mutation ratio r/m was obtained by comparing the non-synonymous SNPs acquired through recombinations relative to point mutations. The SNPs acquired through point mutations are defined as the singleton SNPs associated with SI or ARF, which cannot be categorized to any SNP clusters and have a low level of linkage disequilibrium (*D*′<0.7, with *D*′ defined by Lewontin^60^) with the neighboring SNPs within SNP clusters. A more thorough estimation was also performed using ClonalFrame^41^ by averaging over 15 randomly selected aligned genomic regions of lengths 2000–9000 bp (Supplementary Fig. S8 and Table S2).

## Additional Information

**How to cite this article**: Bao, Y.-J. *et al*. Phenotypic differentiation of *Streptococcus pyogenes* populations is induced by recombination-driven gene-specific sweeps. *Sci. Rep*. **6**, 36644; doi: 10.1038/srep36644 (2016).

**Publisher’s note**: Springer Nature remains neutral with regard to jurisdictional claims in published maps and institutional affiliations.

## Supplementary Material

Supplementary Information

Supplementary Dataset 1

## Figures and Tables

**Table 1:** Statistics of intragenic recombination events within and between sublineages.

	ARF^a^ vs. ARF	ARF vs. SI^b^	SI vs. SI	Other^c^vs. Other	Other vs. Skin	ARF vs. Other
Number of genes with intragenic recombination	23	51	40	87	87	68
Number of fragments with intragenic recombination	39	145	101	437	446	289
Mean length of fragments (bp)	525	389	554	466	475	415
Number of fragments involving recent recombination	9	21	33	—	—	—

^a^Strains associated with acute rheumatic fever (or rheumatogenicity);

^b^Strains associated with skin infection;

^c^All strains in the population other than those associated with skin infection or acute rheumatic fever.

**Table 2:** Functional classification of SNP clusters associated with SI or ARF.

Functional classification	Associated with SI		Associated with ARF		*P*-value (SI vs. ARF)
	# of gene loci	# of genes	# of gene loci	# of genes	
Amino Acids and Derivatives	4	9	11	19	0.39
Carbohydrates	9	12	22	42	0.36
DNA Metabolism	10	17	22	43	0.47
Protein Metabolism	11	13	20	28	0.48
RNA Metabolism	8	9	14	14	0.47
Membrane Transport	10	13	10	14	0.09
Cell Wall and Capsule	6	12	4	10	0.08
Cell Division and Cell Cycle	1	1	5	8	0.34
Virulence, Disease and Defense	3	6	14	20	0.12
Resistance to antibiotic and toxic compounds	0	0	7	8	0.05
Cofactors, Vitamins, and Prosthetic Groups	3	4	7	11	0.55
Fatty Acids, Lipids, and Isoprenoids	3	3	5	9	0.54
Nucleosides and Nucleotides	1	3	6	10	0.26
Regulation	2	4	6	7	0.46
Hypothetical protein	5	5	16	16	0.34
Unclassified	16	27	22	31	0.22
Total^a^	92	138	182	280	—

^a^The total number of gene loci is greater than the total number of derived SNP clusters due to the fact that some of the SNP clusters contain multiple genes.
