# Supplementary material for: Phenotypic differentiation of Streptococcus pyogenes populations is induced by recombination-driven gene-specific sweeps
Source: Sci Rep. 2016 Nov 8;6:36644. doi: 10.1038/srep36644 (PMC5099688; doi:10.1038/srep36644)
Supplement: Supplementary Information [file srep36644-s1.pdf]

Phenotypic differentiation of *Streptococcus pyogenes* populations is induced by recombination-driven gene-specific sweeps

Yun-Juan Bao[1], B. Jesse Shapiro[2], Shaun W. Lee[1,3], Victoria A. Ploplis[1,4], Francis J. Castellino[1,4,*]

[1]W.M. Keck Center for Transgene Research, University of Notre Dame, Notre Dame, IN 46556, USA,
[2]Département de Sciences Biologiques, Université de Montréal, Montréal, QC H3C 3J7, Canada,
[3]Department of Biological Sciences, [4]Department of Chemistry and Biochemistry, University of Notre Dame, Notre Dame, IN 46556, USA

*Corresponding author: Francis J. Castellino, W. M. Keck Center for Transgene Research, University of Notre Dame, Notre Dame, IN 46556, USA
Telephone: 574.631.9152; Telefax: 574.631.8017; Email: fcastell@nd.edu

Table S1. Non-synonymous SNPs associated with SI or ARF induced by point mutations.

Genomic location	Associated allele	Non-associated allele	Amino acid change	*P* value	Gene symbol	Function
SNPs associated with SI						
151,311	G	T	Val280Leu	0.0002	*nga*	Nicotine adenine dinucleotide glycohydrolase
191,948	G	A	Glu57Lys	0.0002	*pgi*	Glucose-6-phosphate isomerase
406,569	T	C	Ile99Thr	0.001	-	Hypothetical protein
573,071	A	T	Glu41Val	0.001	*atpH*	ATP synthase delta chain
580,604	T	C	Val19Ala	0.001	*endA*	DNA-entry nuclease (Competence-specific nuclease)
593,884	G	A	Met682Ile	0.001	*addA*	ATP-dependent nuclease, subunit A
870,474	A	G	Ile495Val	0.001	*proX*	L-proline glycine betaine binding ABC transporter protein
1,146,451	A	G	Lys79Glu	0.0002	-	tRNA (adenine37-N(6))-methyltransferase TrmN6
1,641,579	T	C	Asn243Asp	0.0010	*eep*	Intramembrane protease RasP/YluC
1,722,367	G	T	His21Asn	0.0010	*pbp2A*	Multimodular transpeptidase-transglycosylase
SNPs associated with ARF						
9,547	A	A	Ile7Val	0.0001	-	Hypothetical protein
246,571	A	G	Met106Val	0.0004	*rgpG*	Undecaprenyl-phosphate N-acetylglucosaminyl 1-phosphate transferase
385,008	T	G	Ile590Met	0.0001	-	67kDa Myosin-crossreactive streptococcal antigen
578,399	T	C	Met68Thr	0.0004	-	Hypothetical protein
836,881	G	A	Arg111Gln	0.0004	*folC1*	Dihydrofolate synthase / Folylpolyglutamate synthase
874,737	C	T	Val72Ile	0.0004	*apbE*	Similar to thiamin biosynthesis lipoprotein ApbE
1,098,607	G	T	Thr169Lys	0.0001	*graB*	Protein G-related alpha 2 macroglobulin-binding protein
1,099,594	A	G	Ile379Thr	0.0004	*murZ*	UDP-N-acetylglucosamine 1-carboxyvinyltransferase
1,537,947	T	C	Tyr595His	0.0004	*recD*	RecD-like DNA helicase YrrC
1,668,184	A	G	Thr58Ala	0.0000	*pabB*	Para-aminobenzoate synthase, aminase component
1,756,569	T	C	Asp152Gly	0.0004	*pepO*	Neutral endopeptidase O
1,860,002	A	G	Ile76Val	0.0004	*parB*	Chromosome (plasmid) partitioning protein ParB

Table S2. ClonalFrame inference of the recombination specifics and relative contribution of recombinations to point mutations for 15 randomly selected genomic regions.

Region #	Length of regions (bp)	ρ/θ[a]	r/m[b]	Recombination tract length[c] (bp)
1	8,924	6.9	53.2	1,710
2	7,001	14.0	38.7	561
3	8,521	12.0	43.9	276
4	1,771	6.1	13.2	39
5	3,101	8.9	21.3	280
6	4,761	9.7	56.4	1,305
7	3,301	10.8	28.0	1,387
8	5,101	4.4	21.8	670
9	3,661	5.6	20.7	3,232
10	2,551	6.2	19.2	283
11	6,001	8.9	52.6	2,728
12	3,501	8.9	33.7	887
13	2,341	6.7	24.3	1,039
14	3,401	11.2	57.5	2,830
15	5,601	11.7	54.1	4,131
Median	3,661	8.9	34	1,172

a Relative rate of the number of recombinations to point mutations;

b Relative contributions of recombinations to point mutations;

c Average length of the fragments involved in recombination.

A

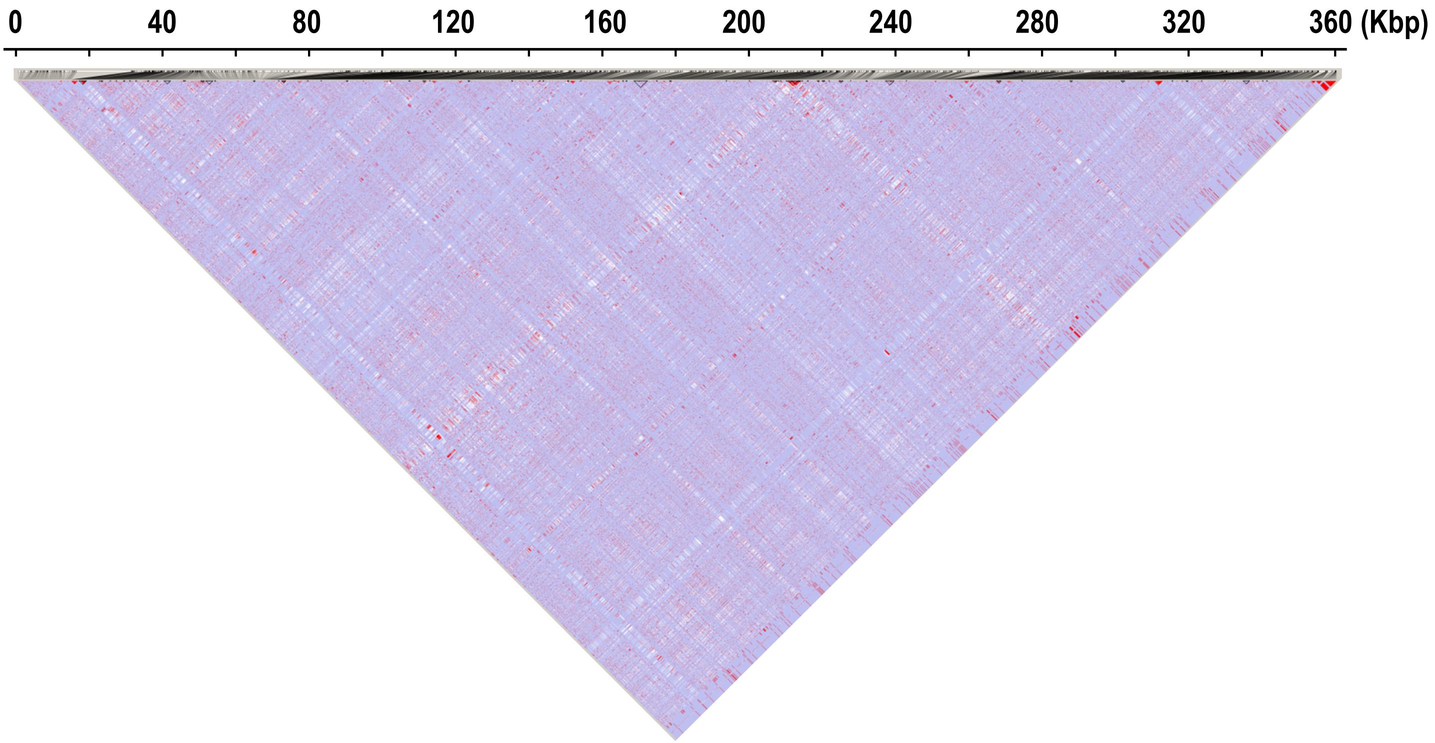

B

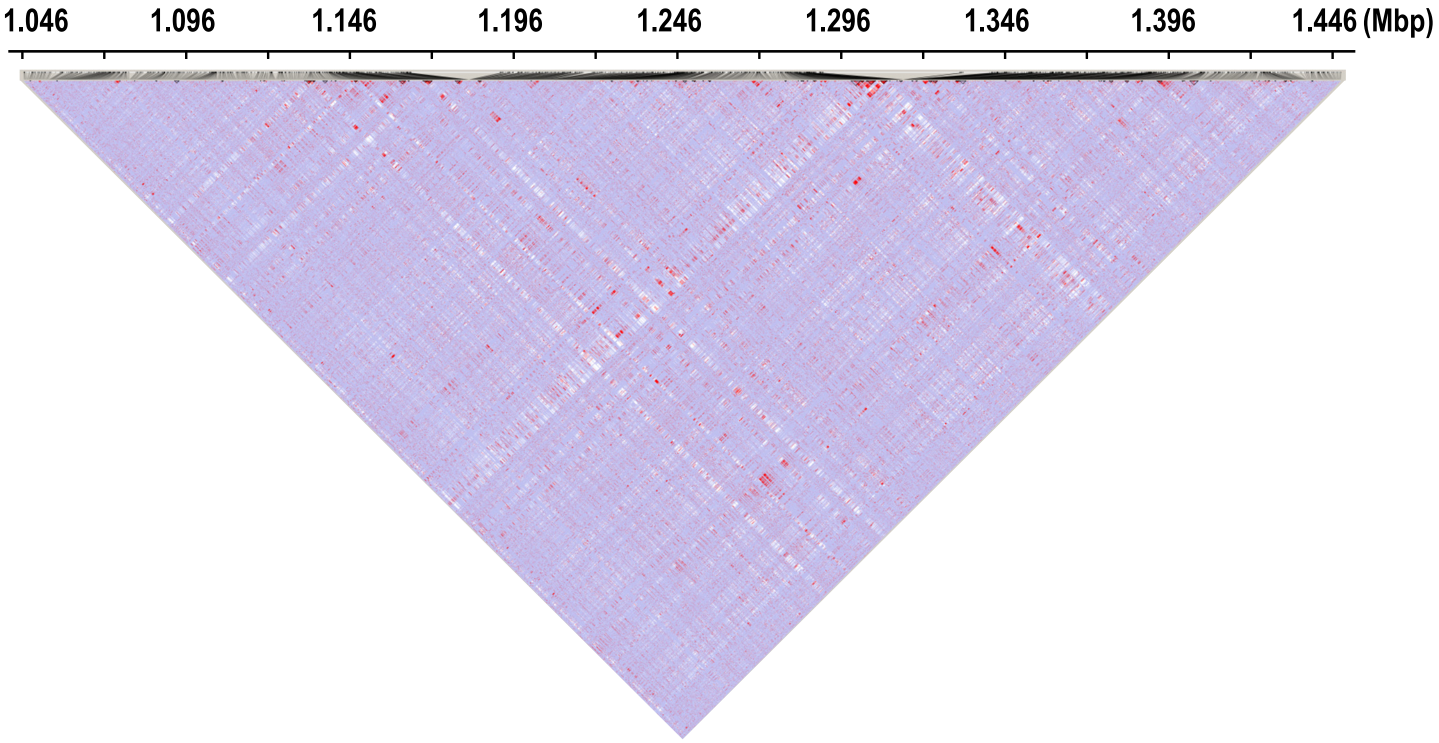

FIG. S1 Patterns of linkage disequilibrium for two representative regions in GAS genomes of length ~ 400 Kbp. (A) LD patterns in the genomic region 1-360,119 bp. (B) LD patterns in the genomic region 1,045,958-1,448,219 bp. The patterns were generated by Haploview based on the pair-wise measure of linkage D' and log of likelihood of odds ratio LOD. The SNP sites with significantly strong LD (D' =1 and LOD > 2) are shown in red, intermediate LD (D' < 1 and LOD > 2) in pink, weak LD (D' < 1 and LOD < 2) in white, and uninformative in purple (D' = 1 and LOD < 2). The neighboring SNPs with strong LD are indicated with red blocks. The x-axis on the top represents the genomic positions of the SNP loci.

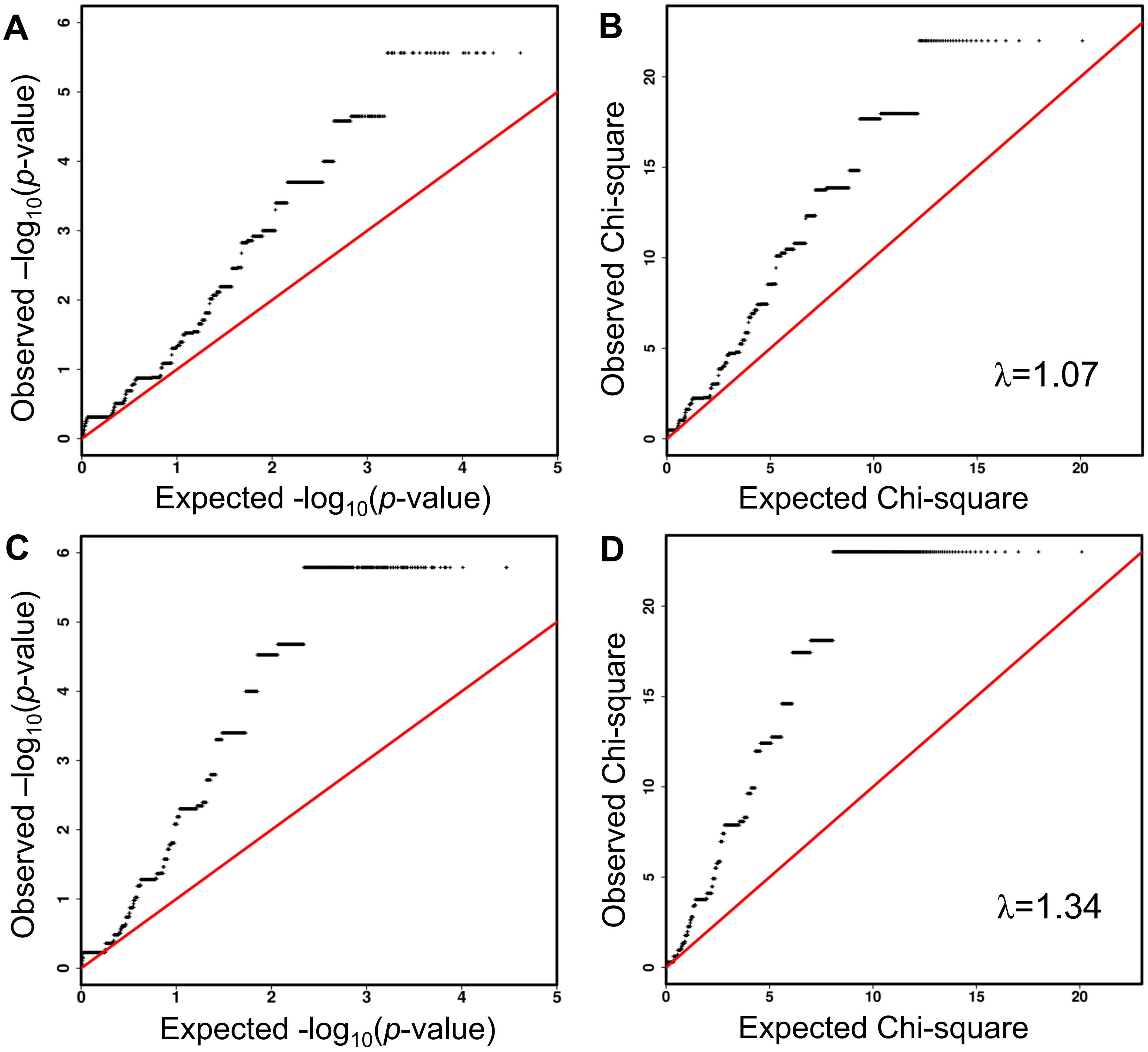

A
B
C
D
Observed –log10(p-value)
Expected -log10(p-value)
Observed Chi-square
Expected Chi-square
λ=1.07
λ=1.34

FIG. S2 Q-Q plots of the association test for the sublineage associated with SI (A,B) and ARF (C,D). The *p*-value (scaled to $-\log_{10}$) and Chi-square are both plotted (in black cross). The diagonal lines are shown in red to indicate the deviation of the observed from expected values. The inflation factors λ for the association test are also shown.

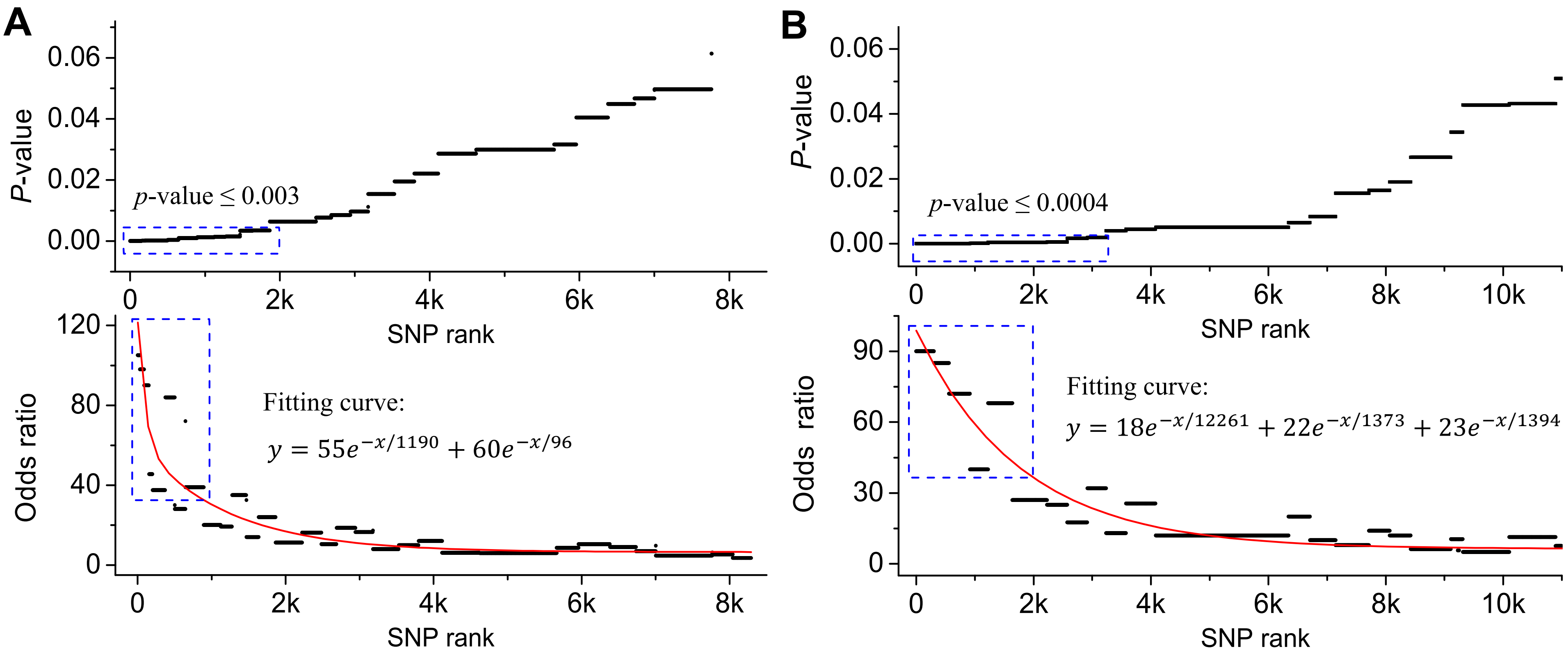

FIG. S3 Distributions of the *p*-value and odds ratio for evaluation of the significant association of SNPs with SI (A) and ARF (B). The SNPs are ranked in the ascending order of *p*-value (top panel) or descending order of odds ratio (bottom panel). The *p*-value was calculated using the Chi-squared test. The SNP is considered to be significant if it falls into the region with the flattest *p*-value curve and the steepest odds ratio curve (framed in blue dotted lines). The steepest regions for odds ratio are determined by fitting the distribution of odds ratio (red lines). The flattest regions for *p*-value are visually determined due to the inaccessibility of automatic fitting. The final set of significant SNPs has a *p*-value ≤ 10-3 and odds ratio ≥ 28.

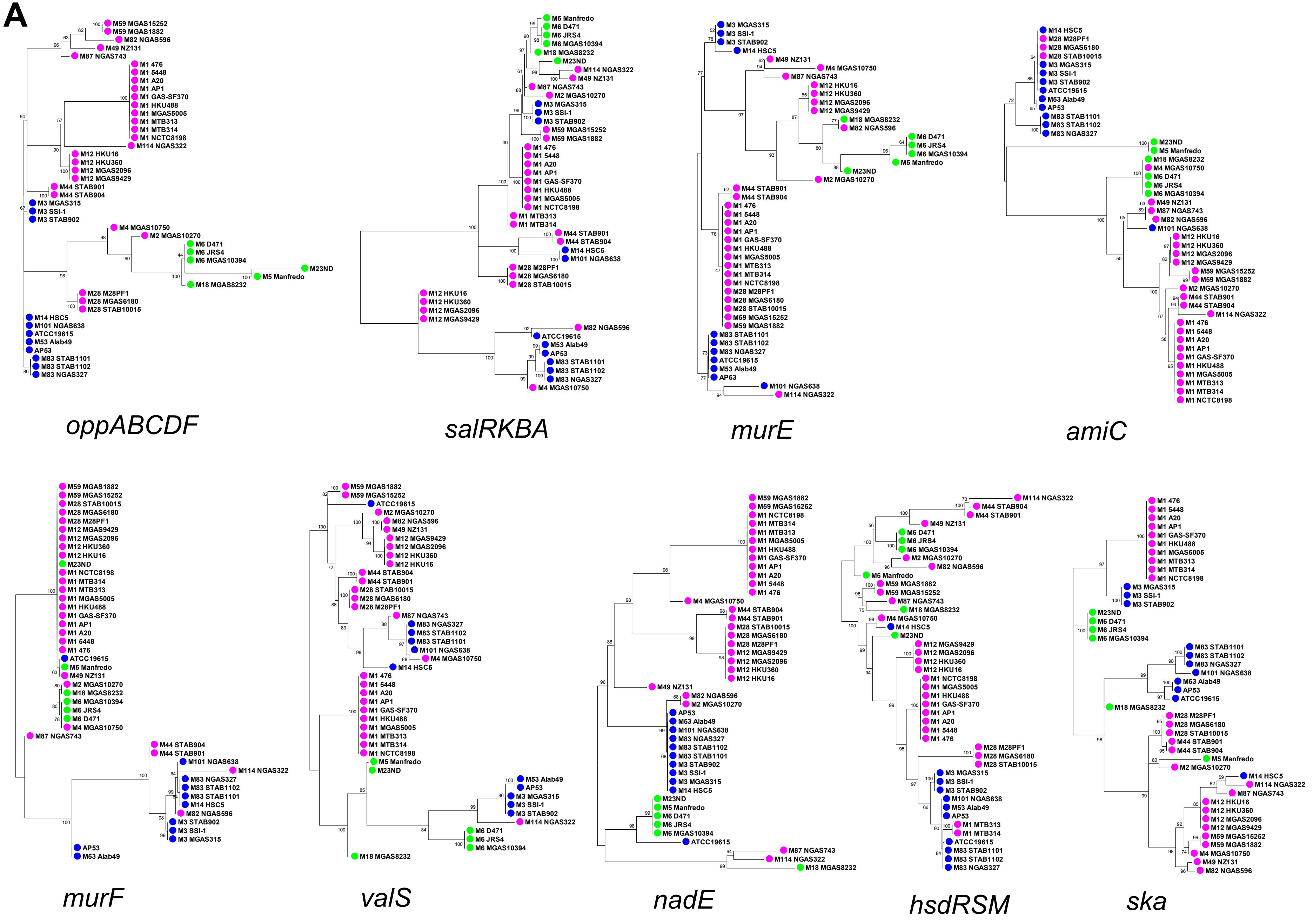
A
oppABCDF
salRKBA
murE
amiC
murF
valS
nadE
hsdRSM
ska

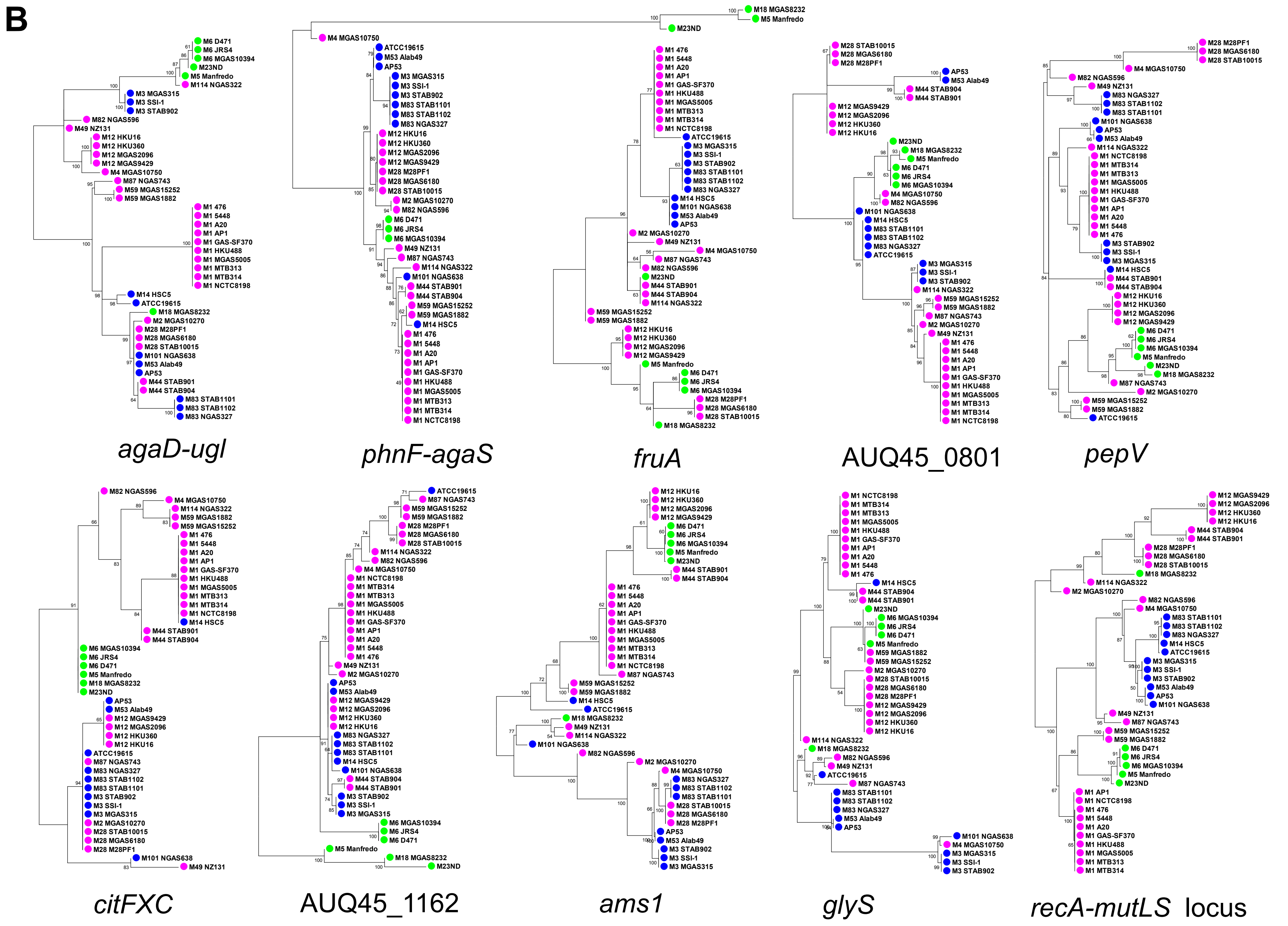
B
agaD-ugl
phnF-agaS
fruA
AUQ45_0801
pepV
citFXC
AUQ45_1162
ams1
glyS
recA-mutLS locus

FIG. S4 Phylogenetic trees reconstructed for SNP loci not identified to be associated with niche-specific infection flanking the associated SNPs. Nine and ten of the gene loci with the most significant SNP clustering associated with SI (A) and ARF (B), respectively were selected. The nodes in the trees are indicated in color: blue for the sublineage associated with SI, green associated with ARF, and magenta for the remaining strains in the population. The trees were built using Neighboring Joining method and optimized based on the model of General Time Reversal (GTR). The support values of the trees were derived by bootstrapping 1000 replicates.

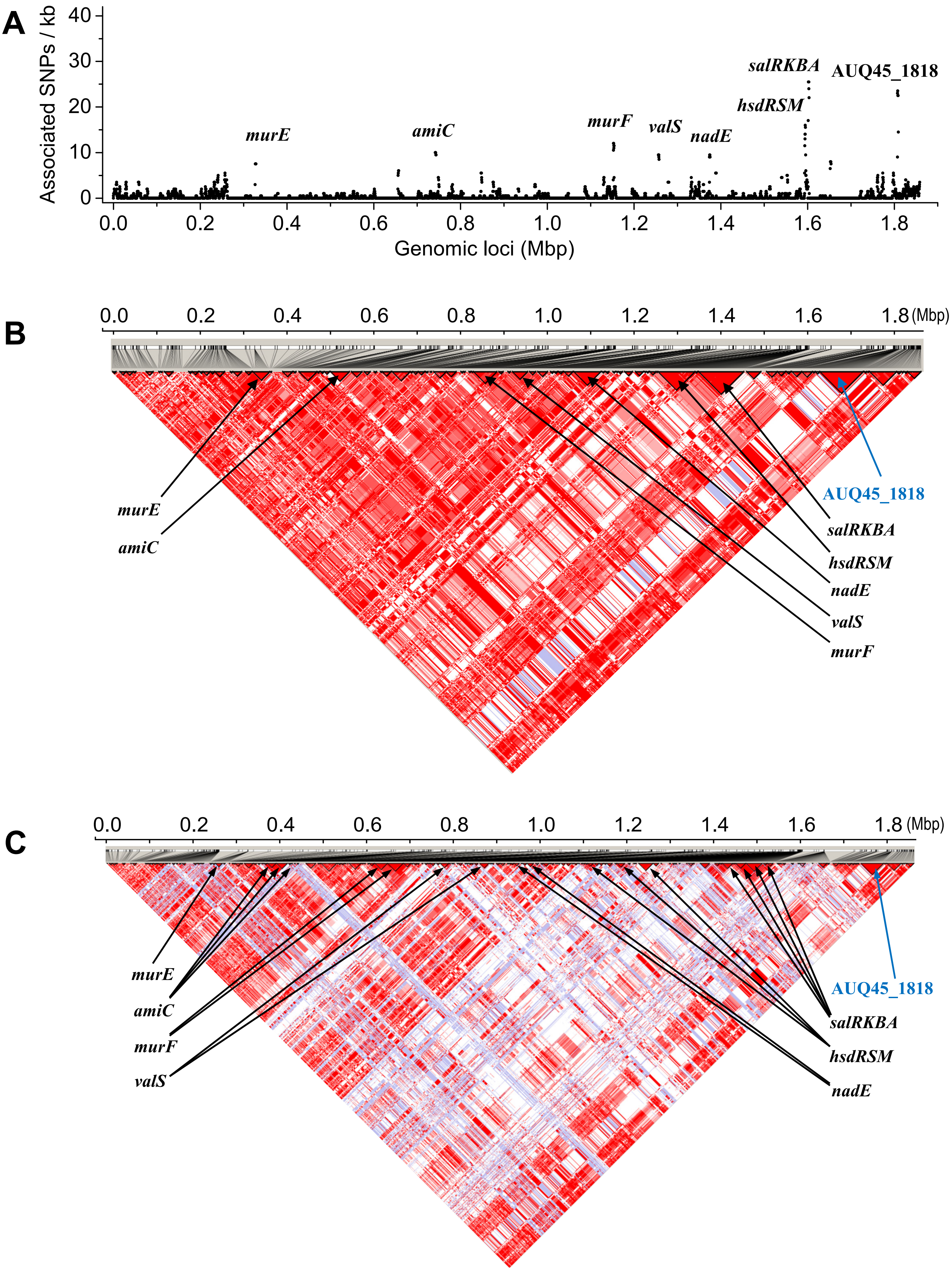
A
Associated SNPs / kb
0
10
20
30
40
0.0
0.2
0.4
0.6
0.8
1.0
1.2
1.4
1.6
1.8
Genomic loci (Mbp)
murE
amiC
murF
valS
nadE
hsdRSM
salRKBA
AUQ45_1818
B
(Mbp)
murE
amiC
AUQ45_1818
salRKBA
hsdRSM
nadE
valS
murF
C
(Mbp)
murE
amiC
murF
valS
AUQ45_1818
salRKBA
hsdRSM
nadE

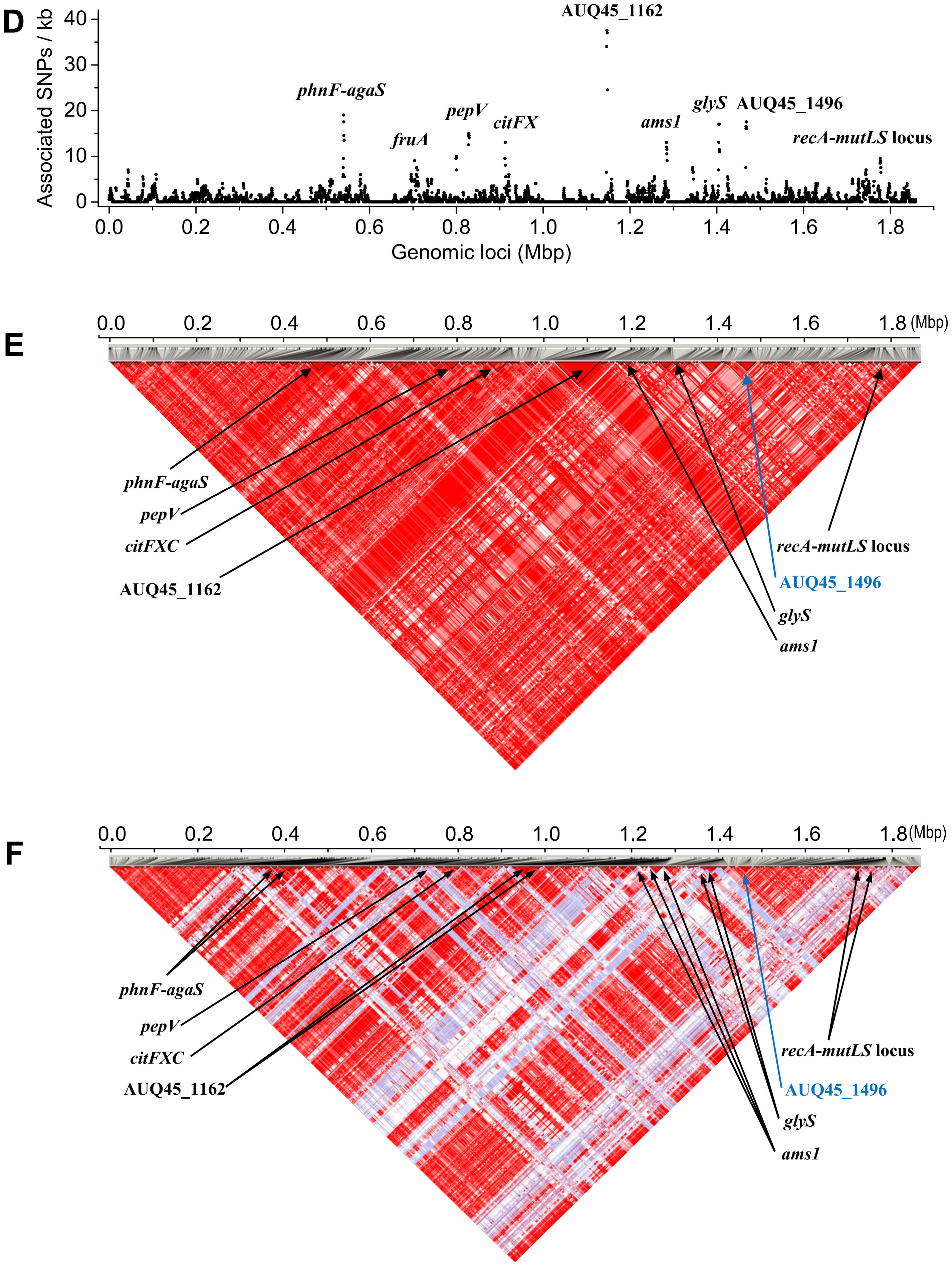

D
Associated SNPs / kb
0
10
20
30
40
0.0
0.2
0.4
0.6
0.8
1.0
1.2
1.4
1.6
1.8
Genomic loci (Mbp)
AUQ45_1162
phnF-agaS
fruA
pepV
citFX
ams1
glyS
AUQ45_1496
recA-mutLS locus
E
0.0
0.2
0.4
0.6
0.8
1.0
1.2
1.4
1.6
1.8
(Mbp)
phnF-agaS
pepV
citFXC
AUQ45_1162
recA-mutLS locus
AUQ45_1496
glyS
ams1
F
0.0
0.2
0.4
0.6
0.8
1.0
1.2
1.4
1.6
1.8
(Mbp)
phnF-agaS
pepV
citFXC
AUQ45_1162
recA-mutLS locus
AUQ45_1496
glyS
ams1

FIG. S5 The changes of the LD patterns for SNPs in associated clusters by adding the nearby SNPs not in association with SI (A,B,C) or ARF (D,E,F). The SNPs were selected from nine and ten of the gene loci with the most significant SNP clustering, the same as in figure S4. The spatial density of SNPs associated with SI in (A) and ARF in (D) is shown for reference. The clustered SNPs are tightly linked with each other with high LD blocks as shown in red triangles in (B) and (E). The tight linkage is interrupted by the nearby SNPs, represented as shorter and fragmented LD blocks in (C) and (F). The SNP sites with significantly strong LD (D'=1 and LOD > 2) are shown in red, intermediate LD (D'< 1 and LOD > 2) in pink, weak LD (D'< 1 and LOD < 2) in white, and uninformative in purple (D'= 1 and LOD < 2), as defined in figure S1. The gene loci with significant changes in the LD pattern are indicated in black. For comparison, two gene loci without additional SNPs and no LD pattern changes are shown in blue.

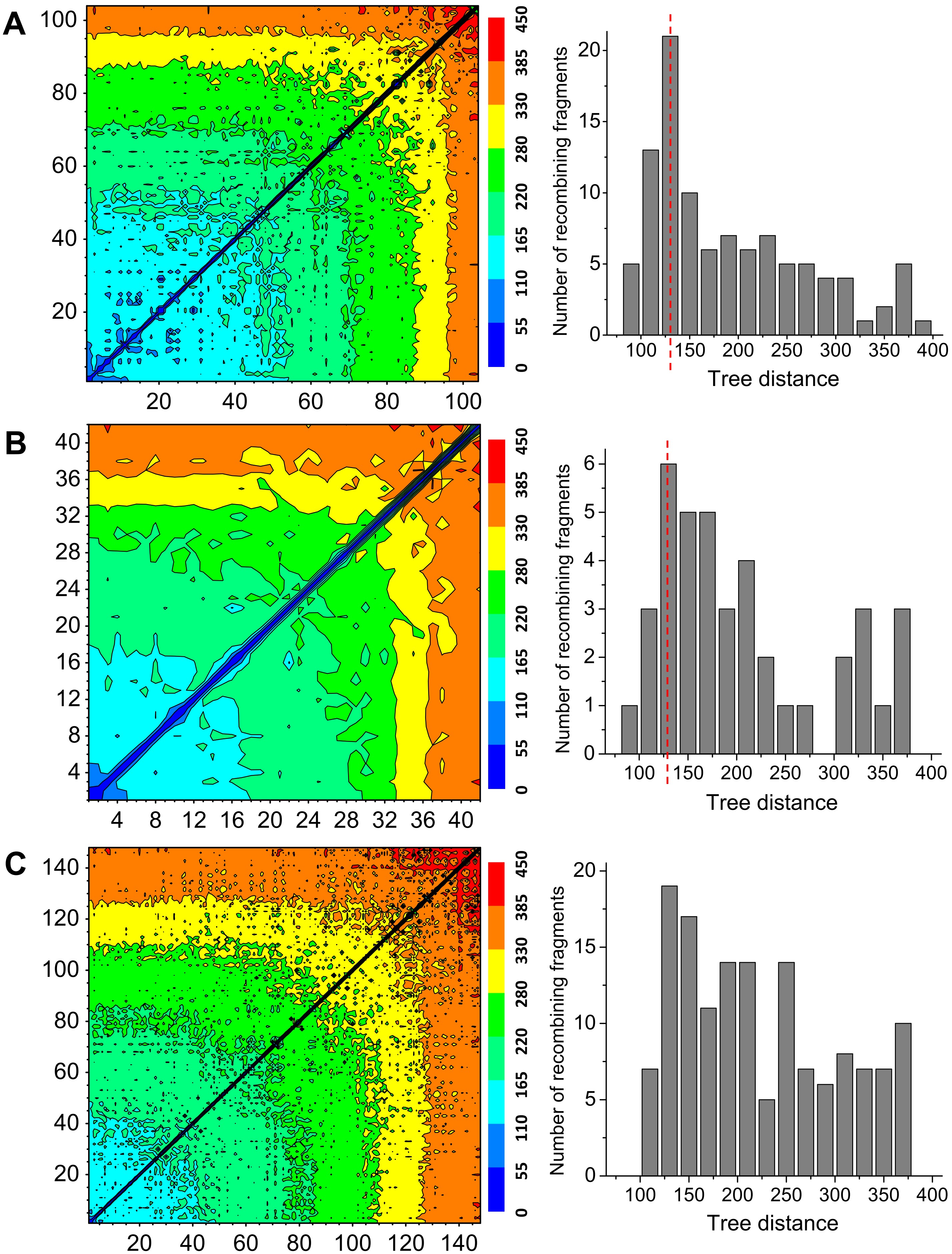

A
B
C
0
55
110
165
220
280
330
385
450
Number of recombining fragments
Tree distance
100
150
200
250
300
350
400

FIG. S6 Profile of the distances of trees for intragenic recombination fragments as compared with the core genome phylogeny. Left panel: heatmap representation of pair-wise distance of trees built for recombination fragments; right panel: distance distribution of the trees built for recombination fragments deviating from the core genome phylogeny. The numbers on the axes show the ranking of the recombination fragments in ascending order of deviation from the core genome phylogeny. Recombination events are classified to three categories: (A) within the sublineage associated with SI; (B) within the sublineage associated with ARF; (C) between the two sublineages associated with SI and ARF. The tree distance is measured using the path difference metric, which calculates the differences in the lengths of the paths for each pair of trees. The color keys for the distances are given on the right side of the heatmaps, For better comparison, the pair-wise distance calculation included three reference trees, *i.e.*, the core genome phylogeny constructed from genome-wide SNPs, the tree from genome-wide coding SNPs, and the tree from combined SNPs associated with SI and ARF (corresponding to the first three elements in the left panel). The three reference trees are more closely related as expected. The red dotted lines indicate the threshold for defining recently emerged recombination events.

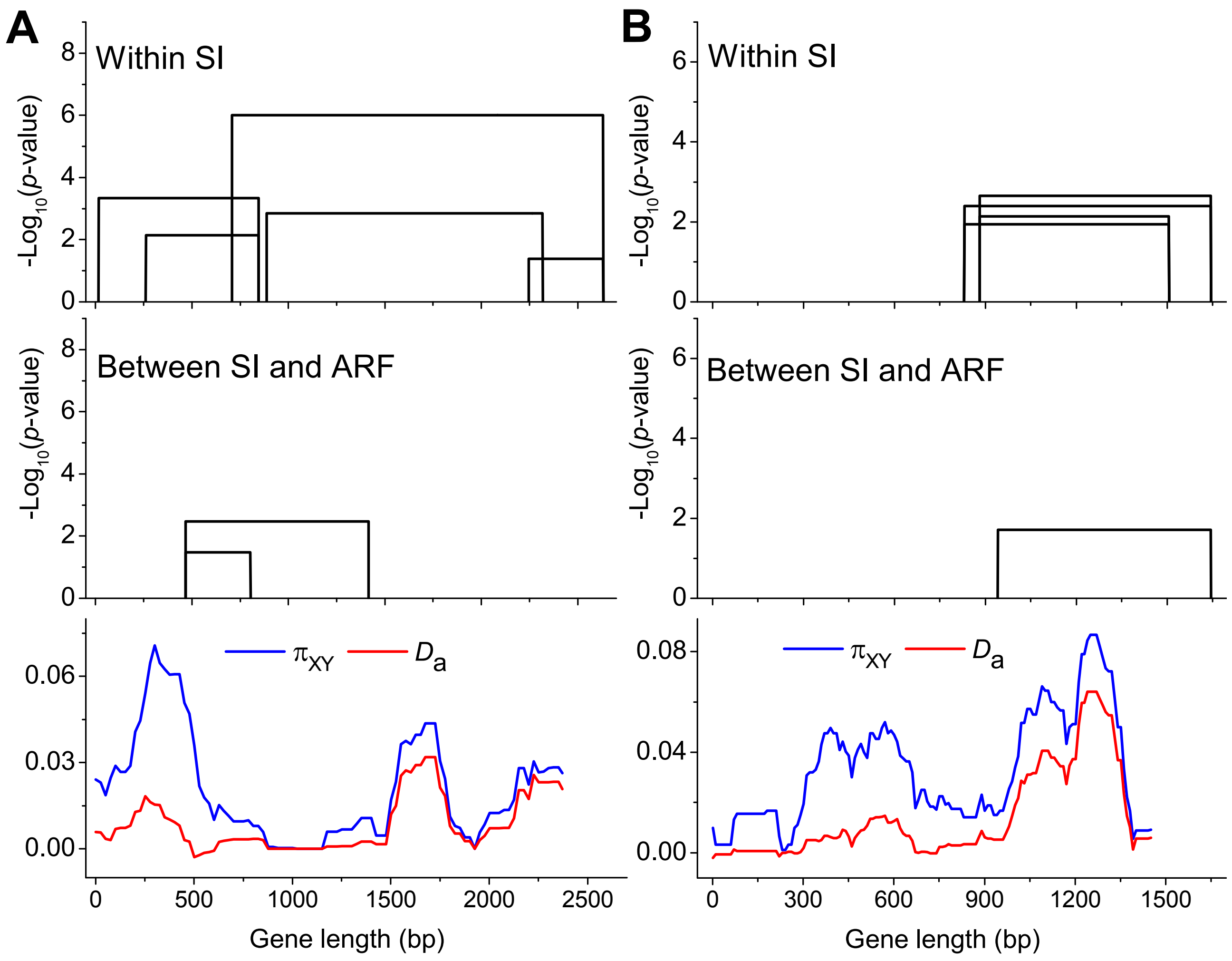

FIG. S7 Graphical representation of recombination events in two example genes *valS* (valyl-tRNA synthetase) and *lacE* (lactose-specific PTS system IIB/IIC component). (A) Recombination events in the gene *valS* within sublineage SI (top panel), and between the sublineages SI and ARF (middle panel), along with the sequence diversity π_{xy} and divergence D_a between SI and ARF (bottom panel). (B) Recombination events in the gene *lacE* within the sublineage SI (top panel), and between the sublineages SI and ARF (middle panel), along with the sequence diversity π_{xy} and divergence D_a between SI and ARF (bottom panel). The recombination fragments are indicated in rectangles. The height of the rectangles is proportional to the confidence of the recombination events, measured as -log10 of the Bonferroni-corrected KA *p*-value. The x-axis shows the gene length, and the beginning of the genes present in the genome is set as position one. The sequence diversity π_{xy} and sequence divergence D_a between the two sublineages SI and ARF are plotted along the gene length.

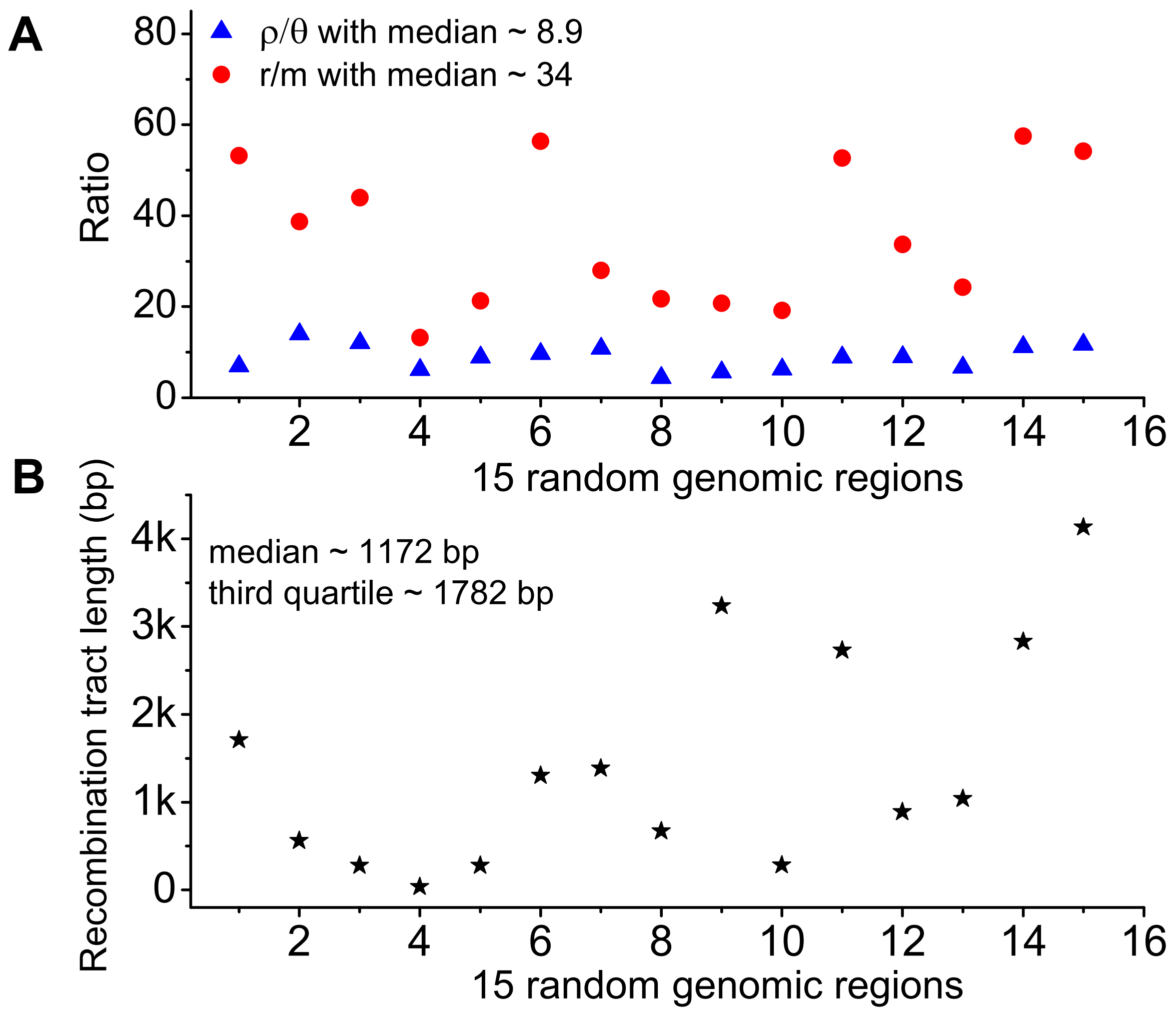

FIG. S8 Inference of genetic recombination using ClonalFrame. (A) Estimation of the relative contributions of recombination as compared with mutation r/m, and relative rate of recombination and mutation ρ/θ. (B) Estimation of recombination tract lengths. The inference was obtained by averaging over 15 randomly selected genomic regions of lengths 2000-9000 bp in GAS genomes.
